# Uncovering Heterogeneity in the Measurement of Psychological Well‐Being in Non‐Western Culture: A Latent Profile Analysis of Ghanaian Undergraduates

**DOI:** 10.1002/brb3.71216

**Published:** 2026-01-28

**Authors:** Daniel William Essel, Frank Quansah, Simon Ntumi, Frank Henry Bonsi, Lawrence Sakyi Larbi, Abdul‐Razak Ishaaq

**Affiliations:** ^1^ Department of Educational Foundations University of Education Winneba Ghana; ^2^ Mathematics and ICT Department Presbyterian College of Education Akropong Ghana

**Keywords:** eudaimonic well‐being, hedonic well‐being, latent profile analysis (LPA), psychological well‐being, university students

## Abstract

**Purpose:**

Psychological well‐being among university students is often examined using variable‐centered approaches that assume population homogeneity. Using Ryff's eudaimonic model and a person‐centered analytic framework, this study examined latent profiles of psychological well‐being among Ghanaian undergraduates, offering insight into how the Western‐derived model functions in a non‐Western cultural context.

**Methods:**

A cross‐sectional design was employed to sample 574 regular undergraduate students from a public university in Ghana. Students completed the 18‐item Ryff's Psychological Well‐Being Scale. Latent profile analysis (LPA) followed by Chi‐square tests were performed using JAMOVI statistical software.

**Findings:**

Four distinct profiles emerged: fully flourishing students (38.7%), harmonious life seekers (45.1%), purposeful self‐actualizers (7.5%), and aspiring actualizers (8.7%). The profiles differed primarily in levels of autonomy, personal growth, and environmental mastery. Well‐being profile membership was not associated with gender but varied significantly by age, although the effect size was small.

**Conclusions:**

The study findings suggest meaningful heterogeneity in eudaimonic well‐being among Ghanaian undergraduates and highlight the importance of culturally sensitive, profile‐based mental health interventions beyond demographic assumptions.

## Graphical abstract

Using latent profile analysis, this study identified four distinct psychological well‐being profiles among Ghanaian undergraduates, demonstrating heterogeneous configurations of eudaimonic well‐being and showing that profile membership varies beyond simple gender and age‐based explanations.

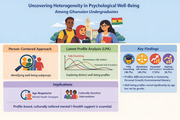

## 1 INTRODUCTION

Psychological well‐being has become a central construct for psychologists, educators, and mental health professionals because of its critical role in human flourishing, mental health, and overall life satisfaction (VanderWeele 2017; Sirgy 2021). Owing to its conceptual complexity, well‐being has been theorized primarily from hedonic and eudaimonic perspectives, each emphasizing different dimensions of positive functioning (Pavot and Diener 2008; Ryff 2014; Ryff 1898). The hedonic approach emphasizes subjective experiences of pleasure, happiness, and life satisfaction, focusing on the balance between positive and negative affect (Bradburn 1969; Diener et al. 1985; Pavot and Diener 2008). This view suggests that well‐being encompasses seeking pleasure and avoiding pain (Rahmani et al. 2018). In contrast, the eudaimonia perspective centers on optimal psychological functioning, personal growth, and the realization of human potential (Ryff 2014).

Although these perspectives differ conceptually, empirical evidence suggests that hedonic and eudaimonic well‐being are positively related and jointly contribute to overall psychological functioning (Diener et al. 2018). Consequently, multidimensional measures grounded in eudaimonic theory such as Ryff's Psychological Well‐Being Scale, have been widely used to represent deeper aspects of human flourishing beyond momentary affect (Ryff and Keyes 1995; Ryff et al. 2021). Ryff's framework for assessing psychological well‐being is one of the most frequently used tools, providing a structured approach to understanding well‐being across six dimensions. These include: (1) Autonomy, which places emphasis on a person's abilities such as self‐determination, independence and the regulation of one's behavior out of the person's free will; (2) Environmental mastery, which refers to a person's ability to manage and control external environment, take advantage of opportunities provided by the environment through physical or mental activities; (3) Personal growth, which refers to the sense of growth wheren an individualdo not only strive to achieve their fixed goals but continues to expand thier potentials; (4) Positive relationships with others, which is understood as having warm, trusting and meaningful relationships with others as well as showing love and empathy to all; (5) Purpose in life, which refers to one's goals, objectives, intentions and sense of direction that creates meaning to life; and (6) Self‐acceptance, which refers to having positive attitude towards one's self and past life and ultimately embracing ones strengths and weaknesses (Ryff et al. 2021).

The transition to higher education is frequently accompanied by academic demands, financial pressures, and social adjustments that place students at heightened risk of psychological distress (Mulaudzi 2023; Cage et al. 2021). Within this context, psychological well‐being has been shown to influence academic engagement, social functioning, and overall life satisfaction, making it a critical resource for student success (Quansah, Srem‐Sa et al. 2024, Quansah, Ankomah et al. 2023). Empirical evidence consistently links psychological well‐being to key academic and social outcomes in university settings. Academically, psychological well‐being has been shown to mediate the relationship between academic stressors (such as intolerance of uncertainty) and academic life satisfaction (Akkoç et al. 2025), while academic satisfaction itself contributes meaningfully to students’ subjective well‐being (Zalazar‐Jaime et al. 2022).

Although higher levels of psychological well‐being are generally associated with improved academic performance (Azmi et al. 2022), findings are not entirely consistent, suggesting that well‐being may operate differently across student subgroups (Klapp et al. 2024). Socially, psychological well‐being supports students’ capacity to form meaningful relationships and maintain emotional stability. Perceived social support and campus connectedness buffer psychological distress and enhance satisfaction with the academic experience (Pidgeon et al. 2017). In the Ghanaian context, monetary resources have been shown to condition the relationship between subjective social status and subjective well‐being (Quansah, Agormedah et al. 2023).

### 1.1 Psychological Well‐Being and Gender Differences

The literature reviewed have revealed mixed results regarding the relationship between psychological well‐being and gender (Ferguson and Gunnell 2016; Katsantonis 2020; Yoon et al. 2023). While some studies report gender differences in psychological well‐being with females generally exhibiting positive outcomes, these findings are not uniform across samples or well‐being dimensions (N‐yelbi and Awuku‐Larbi 2024; Matud et al. 2022).

Beyond student samples, evidence from occupational settings similarly suggests that gender differences may be dimension‐specific rather than global. In a study of lecturers, Yudiani et al. (2024) found significant gender differences across Ryff's dimensions, with men scoring higher on autonomy and self‐acceptance. In contrast, women scored higher on personal growth and positive relationships with others. Relatedly, Li et al. (2015) reported that women demonstrated significantly lower autonomy but greater environmental mastery than men, and that the association between environmental mastery and self‐acceptance was stronger among men. Among college students, Waghmare (2017) also reported gender differences, indicating that men and women may vary in particular components of psychological well‐being and, in some contexts, in overall levels as well. However, not all studies support these patterns. While Högberg (2023) reported contradictory or negligible gender differences in a sample of flood victims, Salleh and Mustaffa (2016) found no gender differences in psychological well‐being and related dimensions (e.g., autonomy, environmental mastery, positive relationship with others, self‐acceptance, and personal growth). In the same vein, Matud et al. (2022) argued that even when gender differences appear on specific dimensions, overall levels of psychological well‐being may not differ meaningfully between men and women.

These inconsistencies suggest that gender effects on psychological well‐being may be context‐dependent and dimension‐specific rather than universal. Importantly, variable‐centered approaches that rely on mean‐level gender comparisons may misrepresent heterogeneity in how well‐being dimensions combine within individuals, particularly in a non‐Western context where communal norms shape social roles and coping resources. This understanding underscores the need for analytic approaches that move beyond simple gender contrasts to examine patterned configurations of well‐being.

### 1.2 Psychological Well‐Being and Age

Age has long been examined as an important demographic factor associated with psychological well‐being, yet empirical findings remain mixed and context‐dependent (Sirgy 2021). Some studies have found systematic age‐related patterns in psychological well‐being, particularly across adulthood. For example, using longitudinal and cross‐sectional data, Springer et al. (2011) found that specific dimensions of Ryff's psychological well‐being (i.e., autonomy and environmental mastery) tend to increase with age, reflecting accumulated life experience and improved emotional regulation. Similarly, Whitman et al. (2024) reported that older adults experienced higher emotional well‐being, with mixed emotions contributing positively to psychological health, whereas younger adults exhibited comparatively lower psychological well‐being.

In contrast, other studies emphasize that age‐related differences in psychological well‐being are neither linear nor deterministic. Drawing on large‐scale and event‐based data, Haehner et al. (2024) demonstrated that individual life experiences, such as exposure to negative or disruptive life events, often exert a more substantial influence on changes in subjective well‐being than chronological age alone. Supporting this view, Li et al. (2023) used data from the Chinese General Social Survey and observed a non‐linear pattern in social well‐being, with lower levels reported in early adulthood, followed by improvement in later age groups. These suggest that the age effect reflects broader socio‐developmental and contextual processes.

Further evidence indicates that environmental and personal factors may outweigh age‐related influences on psychological well‐being. Herrera and Cabrera‐Barona (2022) found that perceptions of environmental stressors, such as air pollution and noise, significantly shaped subjective well‐being, health, and life satisfaction across age groups. Similarly, earlier research has highlighted the role of personality traits, coping strategies, and sensitivity to environmental stimuli in shaping well‐being trajectories (Archontaki et al. 2013; Black and Kern 2020). These studies suggest that individuals of similar ages may experience psychological well‐being in markedly different ways depending on contextual environmental and personal circumstances.

The observed non‐linear associations between age and psychological well‐being indicate that age alone provides limited explanatory power. This underscores the need for person‐centered approaches capable of identifying heterogeneous configurations of well‐being that may cut across age groups, particularly within diverse undergraduate populations.

### 1.3 The Current Study

Despite extensive research on psychological well‐being among university students, most studies adopt variable‐centered approaches that assume population homogeneity and limit meaningful individual differences (Aziz et al. 2024; Matud et al. 2022; Alandete 2013; Sandoval Barrientos et al. 2017). The few person‐centered studies on psychological well‐being were conducted primarily among older adults or non‐student populations. For example, Shima and Muto (2024) identified three subgroups of psychological flexibility (high, moderate, low), and Saadeh et al. (2022) reported three well‐being profiles (worst, intermediate, best) among older adults. Similarly, Fang et al. (2025) identified four subgroups of hedonic well‐being. Notably, the person‐centered examination of eudaimonic well‐being remains scarce in African higher education contexts, particularly among undergraduate populations navigating culturally specific academic and social demands.

Despite increasing interest in psychological well‐being among African populations, no study to date has examined how Ghanaian undergraduates cluster into distinct eudaimonic well‐being profiles using Ryff's multidimensional framework. Existing African studies rely predominantly on variable‐centered analyses (e.g., N‐yelbi and Awuku‐Larbi, 2024). Where person‐centered approaches have been used, they have not been applied to undergraduate groups navigating the unique academic pressures, socio‐economic constraints, and cultural obligations characteristic of Ghanaian higher education. Consequently, little is known about whether the six dimensions of Ryff's model, developed in a Western context, combine in similar or culturally distinct ways among Ghanaian students, a non‐Western context.

Furthermore, although universities increasingly implement mental health initiatives, their effectiveness remains limited by a lack of evidence on whether different student subgroups experience well‐being differently. Without a detailed understanding of heterogeneity, institutional interventions may fail to identify or support students who deviate from normative or average patterns of well‐being. This study addresses these gaps by applying Latent Profile Analysis (LPA) to uncover configuration‐based patterns of psychological well‐being among Ghanaian undergraduates. This person‐centered approach allows the identification of distinct subgroups based on students’ response patterns across Ryff's six dimensions. It also enables examination of whether profile membership varies across demographic factors such as gender and age, areas where existing findings are mixed and derived mainly from non‐African or Western samples.

Accordingly, the study was guided by two research questions: (1) What distinct psychological well‐being profiles emerge among Ghanaian undergraduate students based on Ryff's Psychological Well‐Being Scale? and (2) How does the prevalence of these profiles differ by gender and age? Through this approach, the study aims to generate culturally relevant insights that can inform targeted mental health interventions within Ghanaian higher education.

## 2 Method

### 2.1 Participants

This study employed a cross‐sectional research design, with a total of 574 regular undergraduate students from the University of Education, Winneba (UEW), Ghana, serving as participants. Ethical approval was sought from the university's ethical review board (Reference number: DAA/P.1/Vol.1/39). The stratified sampling approach was used, with participants from various faculties and departments, stratified by academic unit and enrolment size, to guide participant selection. Participants for the study included full‐time undergraduate students aged 18 years or older. Distance education students and individuals unwilling to provide consent were excluded. We notified the class representatives via the departmental WhatsApp groups to inform potential respondents and confirm their availability. We followed up on the scheduled dates and times. Announcements were made during students’ regular classroom lectures to seek their consent (orally and by signing the consent section on the instrument) to participate after all ethical issues were addressed to the participants. Nearly all students who were approached consented to participate. Only a few declined, and dropout rate during data collection was negligible. Considering the large student population, this method ensured that a substantial number of participants were reached while maintaining the heterogeneity necessary for meaningful LPA (Abbott et al. 2010). Participants were recruited based on their enrollment in regular undergraduate programs and their willingness to provide informed consent. A sufficient sample size was determined based on recent latent profile studies (Appianing et al. 2025; Bai and Bai 2025), ensuring statistical power and representativeness.

Out of the total number of 574 participants, 361 (62.9%) were males, and 213 (37.1%) were females (see Table 1). Their ages ranged from 18 to 36 years. Participants aged 31–35 (51.6%) constituted the majority group, while a few participants, 10 (1.7%), were below 19 years. Other participants were aged 20–25 years (110, 19.2%), 26–30 years (19.7%), and 36 years and above (45, 7.8%) (see Table 1). The unusual age distribution of undergraduate students demonstrates the multiple entry routes into the programs for the selected university. Candidates who already have a 3‐year Diploma in Education are admitted through the post‐diploma route into the Bachelor's degree in Education. This cohort often enters the programme in their 30s, thereby contributing to the high mean and maximum ages observed within the sample (Quansah et al. 2025). The presence of such candidates emphasizes the programme's role in accommodating both traditional entrants and those pursuing further professional development after initial qualification.

**TABLE 1:** Demographic information.

Variable	Levels	Count	Percentage
**Gender**	Male	361	62.9
	Female	213	37.1
**Age**	19 years below	10	1.7
	20–25 years	110	19.2
	26–30 years	113	19.7
	31–35 years	296	51.6
	36 years and above	45	7.8

### 2.2 Instrument

Ryff's Psychological Well‐Being Scale (Ryff and Keyes 1995) was adopted for this study. The scale has 18 items across six dimensions: autonomy, environmental mastery, personal growth, positive relations with others, purpose in life, and self‐acceptance, based on the eudaimonic conceptualization of well‐being. Each dimension consists of three items on a 7‐point Likert scale (1 = strongly agree, 2 = somewhat agree, 3 = a little, 4 = neither agree nor disagree, 5 = a little disagree, 6 = somewhat disagree, 7 = strongly disagree).

Items (1, 2, 3, 8, 9, 11, 12, 13, 17, and 18) were reverse‐coded. Some of the questions on the scale include: I have confidence in my own opinions, even if they are different from the way most other people think' (autonomy); “In general, I feel I am in charge of the situation in which I live” (environmental mastery); “I gave up trying to make big improvements or changes in my life a long time ago” (personal growth); “People would describe me as a giving person, willing to share my time with others” (positive relations with others); “I live life one day at a time and do not really think about the future.” (purpose in life); “In many ways I feel disappointed about my achievements in life.” (self‐acceptance). A higher score on the scale indicates a greater level of overall psychological well‐being and vice versa.

Some practical and methodological considerations guided the choice of the 18‐item Ryff's Psychological Well‐Being Scale. In resource‐constrained settings, lengthy instruments can lead to respondent fatigue and reduced attention, compromising data quality (Rolstad et al. 2011). The short form offers a pragmatic solution by minimizing participants’ burden while still capturing the core dimensions of eudaimonic well‐being (Abbott et al. 2010). This balance between brevity and conceptual coverage was essential for ensuring smooth administration.

Moreover, its adoption allowed the researchers to situate the findings within a broader global discourse on psychological well‐being. It is worth noting that the reduced item count (3 items per dimension) may limit internal consistency in some subscales, as shorter scales often yield lower reliability due to restricted item coverage and construct underrepresentation (Stefana et al. 2025). However, the scale's widespread use and theoretical grounding support its continued relevance in comparative studies (Garcia et al. 2023)

The reliability of the overall construct and its six dimensions was estimated using McDonald's Omega, with both Pearson and Polychoric correlations. The estimation based on the Pearson correlation yielded an overall reliability estimate of 0.70 with the following estimates for the sub‐scales: autonomy (0.51), environmental mastery (0.44), personal growth (0.60), positive relations with others (0.40), purpose in life (0.51), and self‐acceptance (0.54). The reliability estimation based on the Polychoric correlation showed a modest improvement with an overall reliability estimate of 0.83 and dimensions yielding the following: autonomy (0.71), environmental mastery (069), personal growth (0.79), positive relations with others (0.65), purpose in life (0.72), and self‐acceptance (0.73). Although some of the dimensions showed moderate reliability estimates, the overall internal consistency was sufficient (Pallant 2020).

The moderate reliability estimates for the scale's domains, even across different estimation procedures, are consistent with reports from other validation studies (Aryani and Umar 2022; Wilson Fadiji et al. 2019). This variation suggests that these dimensions naturally exhibit greater variability due to the complexity of psychological traits and the cultural dynamics that shape individual differences in responses. A scoping review of well‐being measures found that many scales were primarily validated in Western contexts, resulting in lower reliability when applied to non‐Western populations (Zhang et al. 2024). In the Ghanaian context, studies have shown that certain dimensions exhibit lower reliability due to cultural variations in how well‐being is conceptualized. For example, Wilson Fadiji et al. (2019) found that both hedonic and eudaimonic well‐being measures were not clearly distinguished in Ghanaian samples, suggesting that Western‐developed scales may not fully capture local perspectives on well‐being.

The Ryff's Psychological Well‐Being Scale was administered in English Language, the official language of instruction in the Ghanaian education system, from basic to university education. In this regard, no translation was required for the administration of the instrument. It is common for English‐language psychological scales to be developed, calibrated, and used in Ghanaian higher education research due to students’ proficiency (Quansah et al. 2022, 2024; Quansah, Agormedah et al. 2024). Nonetheless, cultural adaptation considerations remain significant, as some domains of the scale may manifest differently within collectivist cultures.

### 2.3 Statistical Analyses

The data obtained from respondents were scrutinized to ensure they were clean for analysis by conducting frequency and percentage counts for all variables to identify any inconsistent responses. Furthermore, the mean, standard deviation, skewness, and kurtosis were computed to describe the data. Pearson product‐moment correlation was performed to examine how the dimensions of Ryff's psychological well‐being scale relate to one another.

The composite scores for each of the six dimensions of psychological well‐being were computed in SPSS and subsequently exported into JAMOVI (version 2.6.2.6) for the LPA. LPA was conducted to address the first research question, which sought to identify distinct subgroups of students based on their patterns of responses across the well‐being dimensions (Ferguson et al. 2020). Although some subscales exhibited moderate reliability, prior simulation and methodological research demonstrate that LPA remains robust under such conditions, particularly when classification precision is high, and class separation is adequate (Masyn 2013; Tein et al. 2013). Therefore, the moderate internal consistency observed in certain dimensions was not expected to compromise the stability or interpretability of the resulting profiles.

Model estimation began with a two‐class solution and progressed sequentially up to a five‐class model. A one‐class model was not considered because the purpose of LPA is to detect latent heterogeneity; a single class would assume complete homogeneity in the population and therefore contradict the foundational logic of mixture modeling (Collins and Lanza 2010; Nylund et al. 2007). Model fit was evaluated using a combination of statistical indices, including the Akaike Information Criterion (AIC), Bayesian Information Criterion (BIC), Sample‐Adjusted BIC (SABIC), and entropy values (Ferguson et al. 2020; Masyn 2013; Tein et al. 2013). These indices were compared across models to determine the most parsimonious and best‐fitting solution. Importantly, the final decision regarding the optimal number of profiles was not based solely on statistical indices but also on theoretical coherence, substantive interpretability, and the meaningful differentiation of psychological well‐being patterns within the student population.

Following the identification of the latent psychological well‐being profiles, we conducted additional analyses to examine whether membership in the profiles differed by gender and age. Because the latent profiles served as categorical outcome variables, the appropriate approach was to test associations between class membership and demographic variables rather than to conduct mean‐level comparisons. Gender was treated as a binary categorical variable (male = 1, female = 0). To determine whether the distribution of males and females differed significantly across the four latent profiles, a Pearson chi‐square test of independence was performed. The chi‐square statistic evaluated whether the observed frequencies of gender within each profile deviated from what would be expected under independence. Effect size was quantified using Cramer's V, which is recommended for nominal‐by‐nominal associations and allows interpretation of the strength of the association irrespective of sample size. Confidence intervals for Cramer's V were also reported to provide a more robust understanding of the effect magnitude.

Age was categorized into five groups (≤19, 20–25, 26–30, 31–35, and ≥36 years) based on the distribution of respondents and the structure of the university's admission system. Because the age variable comprised more than two unordered categories, a chi‐square test of independence was again used to assess whether the proportions of students in each age group differed significantly across the latent profiles. As with gender, Cramer's V was used to evaluate effect size. Given the number of categories, interpretation focused on whether any age‐related differences represented meaningful patterns or merely statistical variations with small practical importance.

## 3 RESULTS

The results from Table 2 indicate a high level of overall psychological well‐being among undergraduate students (Mean = 5.14, SD = 0.769), with varying mean scores across the various dimensions of well‐being. Acceptance was the most prevalent among students (Mean = 5.68, SD = 1.10), followed by personal growth (Mean = 5.61, SD = 1.33). Positive relationships with others were the lowest (Mean = 4.58, SD = 1.303) among the dimensions. The skewness (±2) and kurtosis (±7) values fell within acceptable ranges, indicating that the residuals for each variable's responses were appropriately shaped for statistical analysis without significant distortion (Tabachnick and Fidell 2013).

**TABLE 2:** Descriptive statistics of the six dimensions of psychological well‐being.

			Skewness		Kurtosis	
	Mean	SD	Skewness	SE	Kurtosis	SE
Autonomy	5.18	1.313	−0.422	0.102	−0.187	0.204
Mastery of the environment	4.95	1.173	−0.188	0.102	−0.179	0.204
Personal growth	5.61	1.303	−0.821	0.102	0.393	0.204
Positive relationship with others	4.58	1.327	−0.259	0.102	−0.301	0.204
Purpose in life	4.84	1.369	−0.271	0.102	−0.477	0.204
Acceptance	5.68	1.100	−0.685	0.102	0.128	0.204
Psychological Well‐being	5.14	0.769	−0.175	0.102	−0.738	0.204

### 3.1 Correlations Between the Dimensions of Psychological Well‐Being

**FIGURE 1:**
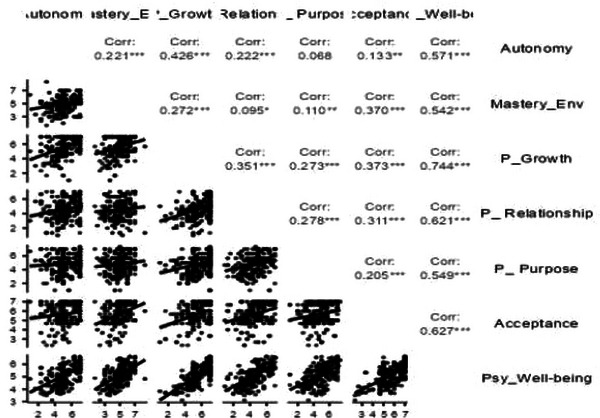
Correlational analysis of the six dimensions of psychological well‐being.

The relationship among the six dimensions of psychological well‐being was explored, as shown in Figure 1. These correlations ranged from 0.068 to 0.744, indicating only positive associations among the dimensions. The association between personal growth and psychological well‐being (*r* = 0.744, *p* < 0.001), for example, was found to be the highest, suggesting the critical role of continuous self‐development in overall well‐being.

Mastery of the environment was positively associated with acceptance (*r* = 0.370, *p* < 0.001). Similarly, purpose in life was positively and significantly associated with a positive relationship with others (*r* = 0.278, *p* < 0.001). However, autonomy and purpose in life were found to be the least correlated and not statistically significant (*r* = 0.068, *p* > 0.05). This might imply that an individual's sense of independence does not necessarily dictate their clarity of purpose. Hence, life meaning might be influenced by broader factors beyond personal autonomy.

### 3.2 Latent Profile Analysis

The four‐class solution model was deemed appropriate for the data, allowing for the examination of class membership. The four‐class solution with entropy value 0.868, Loglik = −5351, AIC = 10,692, BIC = 10,912, and ICL = −11,007 indicates a good balance between model fit and complexity. Although the three‐class model showed a very strong indicator in the entropy value (0.918) and the best ICL value (−10,959), it had a higher AIC = 10,803 and a slightly higher BIC = 10,917 than the four‐class model (see Table 3).

**TABLE 3:** Fit indices for class models.

Classes	LogLik	AIC	AWE	BIC	CAIC	CLC	KIC	SABIC	ICL	Entropy	BLRT (*p*)
2	−5468	10,973	11,232	11,056	11,075	10,937	10,995	10,996	−11,151	0.743	0.009
3	−5376	10,803	11,158	10,917	10,943	10,753	10,832	10,834	−10,959	0.918	0.009
4	−5351	10,769	11,219	10,912	10,945	10,705	10,805	10,808	−11,007	0.868	0.009
5	−5306	10,692	11,239	10,866	10,906	10,614	10,735	10,739	−11,014	0.822	0.009

Although the five‐class solution had the lowest AIC (10,692) and BIC (10,866) across the competing models, it was not retained based on substantive and classification grounds. Notably, the four‑class solution demonstrated higher entropy (0.868) compared to the five‑class model (0.822), indicating clearer classification precision. The bootstrap likelihood ratio test (BLRT) was significant for all comparisons (*p* = 0.009), confirming that additional classes improved fit.

Further substantive interpretability also supported the four‑class solution. As shown in Table 4, the classification matrix revealed strong average posterior probabilities for each class (Class 1 = 0.847, SE = 0.017; Class 2 = 0.959, SE = 0.005; Class 3 = 0.912, SE = 0.009; Class 4 = 0.938, SE = 0.020). All diagonal values exceeded the recommended threshold of 0.80, with low cross‑classification rates (< 0.06), confirming that individuals were clearly assigned to their most likely class (see Table 4). Although Classes 1 (7.5%) and 4 (8.7%) were smaller subgroups, their distinct mean profiles and substantial posterior probabilities (> 0.84) indicate that they are substantively meaningful. The four‑class model balanced statistical adequacy with parsimony, avoiding the risk of over‑fitting associated with the five‑class solution.

**TABLE 4:** Classification matrix for the four‑class solution.

Class	1	2	3	4
1	0.847 (SE = 0.017)	0.027	0.126	∼0.000
2	0.003	0.959 (SE = 0.005)	0.030	0.008
3	0.061	0.028	0.912 (SE = 0.009)	∼0.000
4	∼0.000	0.062	∼0.000	0.938 (SE = 0.020)

**FIGURE 2:**
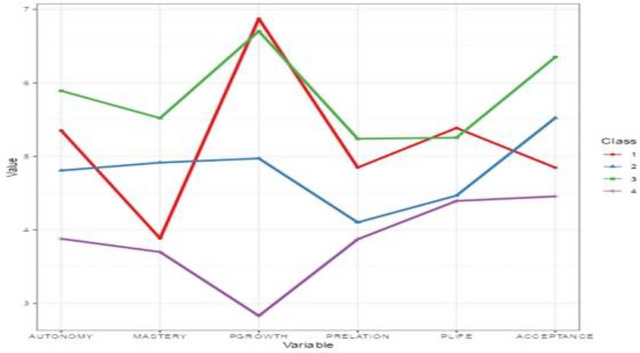
Latent profile line plot of students' response pattern.

The profiles of the four classes were carefully examined and named based on the different responses and scores across the six dimensions of the psychological well‐being scale. The four classes identified were purposeful self‐actualizers, harmonious life seekers, fully flourishing individuals, and aspiring actualizers (see Figure 2).

#### 3.2.1 Class 1: Purposeful Self‐Actualizers (*n* = 43, 7.5%)

This class had the lowest class membership of 7.5%, which demonstrates high levels of personal growth, purpose in life, and autonomy, with mean scores above average on other dimensions. This profile demonstrates strong self‐determination and growth‐oriented tendencies that are consistent with Self‐Determination Theory, which emphasizes intrinsic motivation and competence (Deci and Ryan 2000). Although autonomy is often associated with individualistic cultures, it may be better understood in the Ghanaian context as “relational autonomy.” This is where agency is exercised within the framework of social relationships, cultural norms and values, and communal obligations (Oshana 2020). From the eudaimonic view of well‐being, this class represents individuals who are actively engaged in realizing their potential and viewing themselves as evolving beings seeking to expand continuously. Their high score on autonomy suggests that these students may choose paths aligned with their values rather than external expectations. This may account for their low mean score on environmental mastery. Again, the mean score for the positive relationship with others, which is higher than the average, suggests that students in this class may find nourishment in meaningful social bonds. However, they may not primarily define themselves by them. Relationships are likely to complement their mission rather than be a substitute for it.

#### 3.2.2 Class 2: Harmonious Life Seekers (*n* = 259, 45.1%)

The majority of students fell into this class, comprising 45.1% of the total class membership. This group showed moderate‐to‐high and balanced scores across all dimensions, especially self‐acceptance, personal growth, environmental mastery, and autonomy. This connectedness and self‐acceptance form the foundation of their well‐being. Unlike purposeful self‐actualizers, who emphasize autonomy and growth, harmonious life seekers define themselves basically through acceptance and growth with appreciable social relationships. Their slightly higher personal growth compared to environmental mastery suggests that these individuals are oriented toward ongoing development and self‐improvement, without sacrificing competence in managing their environment. This balance resonates with resilience frameworks, in which adaptive functioning emerges from both accepting limitations and steady progress toward growth (Keyes 2002). The profile, therefore, represents individuals who maintain psychological health by harmonizing acceptance, growth, and mastery of external activities, rather than excelling in a single domain. However, these students may not actively seek growth but maintain psychological equilibrium and interpersonal stability (Linley et al. 2007). Students of this class have a high potential to transition into the higher eudaimonic state if exposed to meaningful challenges.

#### 3.2.3 Class 3: Fully Flourishing Students (*n* = 222, 38.7%)

The third class represents 38.7% of the total class membership. Members of this class consistently showed high levels of well‐being across all dimensions, with outstanding scores in self‐acceptance, personal growth, and environmental mastery. Such students mirror the “optimal functioning” profile in Keyes' mental health continuum (Keyes 2002). This level of flourishing in the continuum denotes that an individual is filled with positive emotion and functions well psychologically and socially. Moreover, this class reflects students who are living in alignment with their values, experiencing life as meaningful and engaging in activities that express their true selves. They are likely to be intrinsically motivated and capable of navigating the complexities of life without losing coherence. For instance, a study conducted among students in Ghanaian public universities using Ryff's multidimensional model of well‐being found that higher scores on the dimensions of well‐being significantly correlated with stronger academic performance (N‐yelbi and Awuku‐Larbi 2024). However, persons who are languishing in life experience low well‐being which may be characterized by emptiness and stagnation, constituting a life of quiet despair that are associated with life full of “hollow,” “empty,” “a shell,” and “a void” (Cushman 1990; Singer 1977) as experienced by students in Profile 4 (aspiring actualizers).

#### 3.2.4 Class 4: Aspiring Actualizers (*n* = 50, 8.7%)

This class constitutes 8.7% of the total class membership. Across the six dimensions of the well‐being scale, students in this group had consistently lower mean scores. The personal growth, autonomy, and environmental mastery dimensions were substantially low, indicating psychological vulnerability among these students. These students may experience a lack of meaning in life, low self‐efficacy, and low sense of purpose and self‐regulation, which are consistent with “struggling” profiles in studies of workplace and student well‐being (Huppert and So 2013; Suldo et al. 2011). This shows a broader perspective on the vulnerability of students within Ghanaian higher education. Mahama et al. (2023) found that during the COVID‐19 pandemic, Ghanaian university students with low mindfulness exhibited diminished academic resilience, poor coping strategies, and reduced self‐efficacy. Although these findings were observed during the COVID‐19 pandemic era, they align closely with the profile of Class 4, whose limited autonomy and mastery suggest reduced capacity to regulate emotions and adapt effectively to academic stressors.

Thus, the struggles of Class 4 members represent the lived realities of students who lack protective psychological resources, leaving them vulnerable to stagnation and distress. Although counselling services are available, Ghanaian students underutilize them due to stigma and cultural barriers, increasing their difficulties in personal growth and environmental mastery (Joana Kyei and Nyarko, 2023)

### 3.3 Differences in the Psychological Well‐Being Profiles by Gender and Age

To determine the differences between the classes identified by gender and age, chi‐square analyses were conducted. The result showed no significant difference across the four classes by gender: χ^2^(3) = 3.91, *p* = 0.271. The effect size index suggested a very weak relationship (Cramer's V = 0.083, 95% CI [0.259–0.276]). This suggests that both male and female students provided similar response patterns across the four classes identified and that membership to any of the classes is not associated with gender. (see Table 5).

**TABLE 5:** Contingency table for psychological well‐being classes by gender.

		SEX		
Membership		FEMALE	MALE	Total
1	Observed	18	25	43
	% within row	41.9%	58.1%	100.0%
	% within column	8.5%	6.9%	7.5%
	% of total	3.1%	4.4%	7.5%
2	Observed	101	158	259
	% within row	39.0%	61.0%	100.0%
	% within column	47.4%	43.8%	45.1%
	% of total	17.6%	27.5%	45.1%
3	Observed	72	150	222
	% within row	32.4%	67.6%	100.0%
	% within column	33.8%	41.6%	38.7%
	% of total	12.5%	26.1%	38.7%
4	Observed	22	28	50
	% within row	44.0%	56.0%	100.0%
	% within column	10.3%	7.8%	8.7%
	% of total	3.8%	4.9%	8.7%
Total	Observed	213	361	574
	% within row	37.1%	62.9%	100.0%
	% within column	100.0%	100.0%	100.0%
	% of total	37.1%	62.9%	100.0%

However, there was a statistically significant association [χ^2^ (12) = 31.8, *p* = 0.001] between the well‐being profiles and age (see Table 6). In other words, the age distribution varied across membership profiles. Conversely, the small size effect (Cramer's V = 0.136, 95% CI [0.002–0.004]) may indicate that age is not the sole determinant of membership, and that the profiles reflect broader patterns of psychological well‐being beyond demographic characteristics.

**TABLE 6:** Contingency table for psychological well‐being classes by age.

Contingency tables							
		AGE					
Membership		19 below	20–25	26–30	31–35	36+	Total
1	Observed	0	13	2	23	5	43
	% within row	0.0%	30.2%	4.7%	53.5%	11.6%	100.0%
	% within column	0.0%	11.8%	1.8%	7.8%	11.1%	7.5%
	% of total	0.0%	2.3%	0.3%	4.0%	0.9%	7.5%
2	Observed	7	50	53	139	10	259
	% within row	2.7%	19.3%	20.5%	53.7%	3.9%	100.0%
	% within column	70.0%	45.5%	46.9%	47.0%	22.2%	45.1%
	% of total	1.2%	8.7%	9.2%	24.2%	1.7%	45.1%
3	Observed	3	32	50	109	28	222
	% within row	1.4%	14.4%	22.5%	49.1%	12.6%	100.0%
	% within column	30.0%	29.1%	44.2%	36.8%	62.2%	38.7%
	% of total	0.5%	5.6%	8.7%	19.0%	4.9%	38.7%
4	Observed	0	15	8	25	2	50
	% within row	0.0%	30.0%	16.0%	50.0%	4.0%	100.0%
	% within column	0.0%	13.6%	7.1%	8.4%	4.4%	8.7%
	% of total	0.0%	2.6%	1.4%	4.4%	0.3%	8.7%
Total	Observed	10	110	113	296	45	574
	% within row	1.7%	19.2%	19.7%	51.6%	7.8%	100.0%
	% within column	100.0%	100.0%	100.0%	100.0%	100.0%	100.0%
	% of total	1.7%	19.2%	19.7%	51.6%	7.8%	100.0%

## 4 Discussion

Using a person‐centered analytic framework, this study identified four distinct psychological well‐being profiles among Ghanaian undergraduates, demonstrating meaningful heterogeneity in the experience of eudaimonic well‐being within this population. The four conceptually distinct profiles are: *Purposeful Self‐Actualizers, Harmonious Life Seekers, Fully Flourishing Students, and Aspiring Actualizers*, each reflecting the variations in students' experiences of well‐being.

The predominance of students identified as harmonious life seekers and fully flourishing students offers a compelling counter‐narrative to prevailing discourses that emphasize adversity in the Ghanaian higher education context. Despite challenges often associated with university life in Ghana, such as resource constraints, shifting pedagogical expectations, and psycho‐social stressors, many students appear to possess adaptive psychological capacities. This observation resonates with a study by Quansah, Ankomah et al. (2023) that emphasizes the buffering role of social and economic support systems in promoting student well‐being. Moreover, the moderate‐to‐high levels of environmental mastery and self‐acceptance among these groups may reflect culturally grounded resilience, which may be fostered through familial bonds, communal coping strategies, or religious faith. This observation is consistent with previous research, which found that African students often draw psychological strength from collectivist cultural norms and shared identity, which enhance emotional stability and perceived life (Salleh and Mustaffa 2016).

The identification of the aspiring actualizers subgroup, representing 8.7% of the sample, raises concern about the psychological well‐being of a notable minority of students. This group exhibited persistently low scores across all measured dimensions of well‐being, indicating elevated psychological vulnerability and potential risk of disengagement from academic and social life. This finding can be situated within a recent report that some undergraduate students at the selected university risk being expelled from school due to low cumulative grade point averages (Agyei‐Lartey 2025). Such students may be struggling to manage the pressures of the academic environment, as indicated by the low mean score in this study (See Figure 2). Similar patterns have been observed in recent African research, which highlights increasing distress among university students exposed to academic pressure, limited institutional support, and insufficient social resources (Dey et al. 2022; Makhubela 2021). These findings suggest that psychological distress may not be anomalous but indicative of broader systemic challenges within higher education.

Another minority group that emerged was the purposeful self‐actualizers, with 7.5% of the total sample. This subgroup represents a psychologically enriched pattern marked by high autonomy, personal growth, and purpose in life. Their profile resembles patterns described in eudaimonic well‐being research, where self‐direction and intrinsic motivation contribute to positive psychological functioning (Ryff et al. 2021). However, their lower environmental mastery suggests potential challenges in navigating institutional structures. This tension between personal agency and environmental constraints aligns with prior research findings indicating that in collectivist educational systems, autonomy‐oriented students may struggle to adapt to rigid institutional processes (Li et al. 2015). Yet, their emergence indicates a growing cohort of Ghanaian youth navigating modern individualistic aspirations while seeking meaning within culturally embedded expectations

The four‐profile solution identified in this research aligns with a growing body of literature demonstrating that psychological well‐being is heterogeneous. Although previous studies have reported varying numbers of profiles depending on the sample and the dimensions assessed, there are notable consistencies with the patterns observed. For instance, the three‐profile structures identified among older adults in Japan (Shima and Muto 2024) and in multi‐country ageing research (Saadeh et al. 2022) similarly distinguished groups characterized by high, moderate, and low well‐being; this mirrors the fully flourishing, harmonious life seekers and aspiring actualizers observed in the current study. Likewise, Fang et al. (2025) identified four subgroups reflecting positive, mixed, and vulnerable well‐being patterns under a hedonic framework, a structure conceptually similar to the present four‐profile solution despite differences in measurement models.

These observed convergences suggest that the differentiation between flourishing, moderately functioning, and psychologically vulnerable groups may reflect a broader pattern observable across cultural and developmental contexts. However, the emergence of purposeful self‐actualizers in the present Ghanaian sample (i.e., characterized by elevated autonomy, purpose, and personal growth, but comparatively lower environmental mastery) highlights a culturally contextual variation that is less evident in Western samples. This profile may reflect unique socio‐cultural tensions experienced in Ghana, where increasing individualistic aspirations coexist alongside collectivist expectations and structural constraints. Thus, while the number and general structure of profiles align with prior research, the specific configuration of the Ghanaian patterns underscores the importance of cultural context in shaping how well‐being dimensions cluster within individuals.

The study found no statistically significant differences in psychological well‐being profile membership between males and females. Although several studies have reported gender variations on specific dimensions of well‐being (N‐yelbi and Awuku‐Larbi, 2024; Matud et al. 2019), other research, including multi‐country and contextualized investigations, similarly reports negligible or inconsistent gender effects (Högberg 2023; Salleh and Mustaffa 2016). The present findings, therefore, align with the literature suggesting that overall psychological well‐being may not systematically diverge by gender, particularly in non‐Western contexts, where social roles, communal expectations, and coping resources may shape well‐being in ways not easily captured by binary gender comparisons. We also acknowledge that the non‐significant gender difference in the well‐being profile could be attributable to limited statistical power to detect subtle associations or to the possibility that gender differences in well‐being manifest through more complex psychosocial processes than can be captured by categorical comparisons.

For age, the analysis revealed a statistically significant association with profile membership; however, the effect size was small, indicating that the practical relevance of this relationship is limited. This finding echoes studies showing that age‐related differences in well‐being are often modest and shaped by broader life experiences rather than chronological age alone (Springer et al. 2011; Haehner et al. 2024). The skewed age distribution in our sample, reflecting the university's multiple‐entry pathways for mature students, may also have constrained variability, contributing to a pattern where age differences reach statistical significance but lack substantive distinction across profiles.

The findings of the study reveal important insights into the Ghanaian cultural and contextual dynamics of psychological well‐being. In Ghanaian society, a strong emphasis is placed on communal relationships, social reciprocity, and interdependence, suggesting that well‐being is often embedded within relational and collective structures. For instance, the relatively lower scores on the environmental mastery and autonomy dimensions in some profiles reflect culturally grounded patterns in which decision‐making and life direction are shaped through family expectations, communal obligations, and respect for authority figures (Quansah 2025). Conversely, the consistently high scores on positive relations and self‐acceptance among the flourishing profiles align closely with Ghanaian values that prioritize harmonious relationships, community belonging, and spiritual resilience. These cultural dynamics help explain why demographic variables such as gender and age show limited substantive influence; psychosocial experiences intertwined with communal norms may exert a more substantial effect on students’ well‐being than demographic categories alone.

While the cultural insights drawn in this study are meaningful, they must be interpreted alongside the psychometric characteristics of the Ryff scale as applied in this context. The modest reliability observed for Environmental Mastery and Positive Relations dimensions suggests that these dimensions may function differently within Ghanaian cultural frameworks. Such measurement variability may influence the sharpness of distinctions between profiles, particularly those characterized by lower overall well‐being. However, the stability of the four‐class solution, supported by high entropy and strong posterior probabilities, indicates that the profiles are not statistical artefacts but represent meaningful patterns of responding even when some dimensions show reduced internal consistency.

At the same time, our findings highlight the need to reflect critically on the applicability of Ryff's conceptual model in African contexts. Constructs such as autonomy and environmental mastery, which are central to Ryff's eudaimonic theory, may manifest differently in cultures that emphasize communal decision making, relational interdependence, and spiritual anchoring. This may partially explain why some subscales yielded lower reliability and why specific dimensions did not strongly differentiate profiles. A refined model of well‐being in African settings may require incorporating relational, communal, and spiritual dimensions that are less central in Western theories but are foundational to Ghanaian conceptions of flourishing. Future work should therefore explore culturally grounded modifications to the model or the development of hybrid frameworks that integrate both universal and culturally specific elements of well‐being.

### 4.1 Practical Implications

The findings of this study have critical implications for how psychological well‐being is experienced within the Ghanaian context, a non‐Western sample. Identifying four latent profiles demonstrates that students differ not only in the level of their well‐being but in the configuration of its underlying dimensions. In an educational setting where universities face increasing pressure to support diverse student populations (i.e., ranging from traditional entrants to mature, working students), profile‐specific strategies are essential for tailoring mental health and academic support in culturally meaningful ways.

For students characterized as purposeful self‐actualizers, stakeholders should prioritize sustaining their momentum by integrating opportunities for advanced academic engagement, leadership cultivation, and structured mentorship. In Ghanaian settings where hierarchical guidance and respect for elders are culturally salient, pairing these students with senior academics or professionals can reinforce their motivation and channel their autonomy toward long‐term contributions to their careers and communities. Additionally, harmonious life seekers, the most prevalent profile, should receive support through strengthening relational resources, peer networks, and collaborative learning structures. Capitalizing on Ghana's collectivist culture, programs that enhance students’ sense of belonging (such as community forums, group‐based resilience workshops, or peer‐support initiatives) can help sustain their well‐being and encourage gradual progression toward higher self‐realization. We recommend that institutional strategies for fully flourishing students aim to maximize their potential through opportunities for service, innovation, and mentorship. They should be encouraged to lead student groups, facilitate peer‐support activities, or participate in community outreach, which aligns with Ghanaian values of communal responsibility and positions them as catalysts for wellbeing within the university ecosystem.

Aspiring actualizers represent the subgroup most in need of targeted support. Their lower functioning across well‐being dimensions calls for multi‐layered interventions, including accessible counselling, emotional skills training, and structured academic guidance. Given the stigma surrounding formal mental health services in Ghana, universities should integrate culturally sensitive approaches, such as faith‐based support systems, strengths‐based coaching, and group mentoring, to normalize help‐seeking. Early identification and proactive engagement are crucial for preventing further academic and psychological decline. We highlight that effective mental health interventions in Ghanaian universities must be culturally grounded, context‐responsive, and differentiated according to students’ psychological configurations. By translating heterogeneity into actionable strategies, institutions can more effectively foster environments that promote both academic achievement and holistic well‐being.

The findings also point to several important directions for future research. First, given the modest reliability of some subscales, there is a need for cultural validation studies to examine whether Ryff's well‐being dimensions fully capture Ghanaian conceptualizations of flourishing, particularly in relation to communalism, spirituality, and social reciprocity. Scale refinement or the development of indigenous well‐being indicators would strengthen conceptual accuracy in future work. Second, because the current study employed a cross‐sectional design, it cannot determine whether the latent profiles identified are stable or whether students transition between profiles over time. Longitudinal research is therefore essential to assess profile stability, understand developmental trajectories, and examine how academic and life events shape movement across well‐being classes.

Third, integrating mixed‐methods or qualitative approaches would provide deeper cultural insight into how students interpret constructs such as autonomy, mastery, and purpose, particularly because these dimensions may carry different meanings in Ghana than in Western contexts. Finally, future studies involving multiple universities would improve generalization and allow comparison of well‐being patterns across diverse Ghanaian educational contexts.

### 4.2 Limitations

While this study provides valuable insights into the heterogeneity of psychological well‐being among Ghanaian undergraduate students, several limitations warrant consideration. First, the use of a cross‐sectional design limits causal inferences regarding the relationships between psychological profiles and demographic or psycho‐social factors. Second, the reliance on self‐reported data may introduce common‐method biases, recall inaccuracies, and social‐desirability effects, particularly in a cultural context where psychological introspection is shaped by communal norms and where mental health discourse may be influenced by stigma (Okello and Musisi 2015). These factors may have contributed to underreporting of distress or overrepresentation of socially valued attributes such as resilience and relational harmony.

Third, although widely used, the Ryff Psychological Well‐Being Scale was developed in Western contexts and may not fully capture Ghanaian or broader African conceptualizations of well‐being. Cultural values emphasizing relational interdependence, communal responsibility, and spiritual grounding may influence how students interpret items related to autonomy or environmental mastery, potentially reducing the cultural sensitivity of these dimensions. In addition, some subscales demonstrated modest internal consistency, which may limit the precision of certain well‐being indicators. Although LPA is generally robust to moderate measurement variability, supported by strong entropy and posterior classification probabilities in this context, the lower reliability of specific subscales should be considered when interpreting the key differences across profiles.

Fourth, the limited generalization of the findings is acknowledged, as the study used a single institution with a sample size of 574 that may not be representative of the broader Ghanaian higher education landscape (Dzakadzie and Quansah 2023). The institution's unique admission structure, which includes a large proportion of mature students entering through post‐diploma pathways, further limits findings generalization. Therefore, interpretations should be made with sensitivity to both the cultural and institutional context.

## 5 Conclusion

This study advances a detailed understanding of psychological well‐being in the Ghanaian higher education context by demonstrating that students’ well‐being reflects a clustered, distinct configuration of functioning. The findings illustrate how different dimensions of Ryff's model combine into culturally embedded ways to shape students’ subjective experiences. The absence of gender differences and the relatively small age effect observed suggest that demographic categories alone are insufficient to explain variations in well‐being in this context. This pattern strengthens the argument that psycho‐social and cultural dynamics (such as communal relational expectations, resource constraints, and institutional pressures) play a more pivotal role than demographic markers in shaping how well‐being is organized among Ghanaian students.

The study also critically contributes to broader debates on the cross‐cultural applicability of Western psychological frameworks in non‐Western contexts. The profile structure, together with the modest performance of some subscales, highlights the need to interrogate how constructs such as autonomy or environmental mastery translate in collectivist, relational, and spiritually grounded contexts. These findings thus support ongoing calls for culturally responsive refinements to eudaimonic models of well‐being and for the development of hybrid conceptual frameworks that more accurately reflect African lived realities. Ultimately, the value of this study lies in its demonstration that culturally grounded, profile‐specific insights can inform more effective mental health strategies in higher education. Universities operating in similar contexts can benefit from interventions that recognize the differentiated psychological needs of students. By integrating person‐centered evidence with cultural awareness, higher education institutions can design policies and practices that foster resilience, belonging, and academic flourishing among diverse student populations.

## Author Contributions

**Daniel William Essel**: conceptualization, methodology, writing – original draft, data collection. **Frank Quansah**: conceptualization, formal analysis, writing – original draft, supervision. **Simon Ntumi**: literature review, writing – review and editing. **Frank Henry Bonsi**: statistical analysis, data visualization. **Lawrence Sakyi Larbi**: literature review, formal analysis, writing – original draft. **Abdul‐Razak Ishaaq**: data curation, editing and formatting. All authors reviewed and approved the final manuscript.

## Funding

The authors have nothing to report.

## Conflicts of Interest

The authors declare no conflict of interest.

## Data Availability

The datasets used and/or analyzed during the current study are available from the corresponding author on reasonable request.
